# PReS-FINAL-2067: How does the self-experienced HRQOL work for children with JIA - juvenile idiopathic arthritis in Stockholm?

**DOI:** 10.1186/1546-0096-11-S2-P79

**Published:** 2013-12-05

**Authors:** K Berggren

**Affiliations:** 1 Astrid Lindgren Childrens Hospital, Karolinska University Hospital, Stockholm, Sweden

## Introduction

The aim of this study was to examine how children/teenagers with JIA - Juvenile Idiopathic Arthritis and its subgroups perceived their HRQOL - Health related quality of Life and to see if there were any correlation between HRQOL, self-rated ability of ADL and the amounts of inflamed joints.

## Objectives

The subjects were 50 children/teenagers, 8-18 years (mean 13,7), 37 girls and 13 boys. 26/50 with Juvenile Polyarthritis, and 30/50 was treated with biological drugs.

## Methods

ADL was measured with CHAQ - Childhood Health Assessment Questionnaire. The amount of joints was assessed by doctors. The HRQOL-instrument used was Disabkids, both the Generic version and the Arthritis version. The Generic version contains 37 questions and the Arthritis version 15 questions.

## Results

Compared to other children with a chronic disease, the HRQOL estimates of the studied group is lower. The study showed a moderate correlation (r = -0.43 - -0.70) between CHAQ and 6 of the subgroups in Disabkids generic and in 1 of 2 subgroups in arthritis - Impact. CHAQ correlated to physical functions but also to mental and social functions. Only a mild correlation was seen between the doctors assessment of active joints (r = 0.31). The participants considered that the questions were important; easy to answer and took < 20 minutes.

## Conclusion

The conclusion is that Disabkids can be used as a complement to other data collection for children with JIA.

## Disclosure of interest

None declared.

